# Effects of avatar shape and motion on mirror neuron system activity

**DOI:** 10.3389/fnhum.2023.1173185

**Published:** 2023-10-04

**Authors:** Yuki Miyamoto, Hirotaka Uchitomi, Yoshihiro Miyake

**Affiliations:** ^1^Department of Systems and Control Engineering, School of Engineering, Tokyo Institute of Technology, Yokohama, Japan; ^2^Department of Computer Science, School of Computing, Tokyo Institute of Technology, Yokohama, Japan

**Keywords:** humanness, avatar, mirror neuron system, μ-wave attenuation, electroencephalogram MNS for perceiving avatars’ humanness

## Abstract

Humanness is an important characteristic for facilitating interpersonal communication, particularly through avatars in the metaverse. In this study, we explored the mirror neuron system (MNS) as a potential neural basis for perceiving humanness in avatars. Although previous research suggests that the MNS may be influenced by human-like shape and motion, the results have been inconsistent due to the diversity and complexity of the MNS investigation. Therefore, this study aims to investigate the effects of shape and motion humanness in avatars on MNS activity. Participants viewed videos of avatars with four different shapes (HumanShape, AngularShape, AbbreviatedShape, and ScatteredShape) and two types of motion (HumanMotion and LinearMotion), and their μ-wave attenuation in the electroencephalogram was evaluated. Results from a questionnaire indicated that HumanMotion was perceived as human-like, while AbbreviatedShape and ScatteredShape were seen as non-human-like. AngularShape’s humanity was indefinite. The MNS was activated as expected for avatars with human-like shapes and/or motions. However, for non-human-like motions, there were differences in activity trends depending on the avatar shape. Specifically, avatars with HumanShape and ScatteredShape in LinearMotion activated the MNS, but the MNS was indifferent to AngularShape and AbbreviatedShape. These findings suggest that when avatars make non-human-like motions, the MNS is activated not only for human-like appearance but also for the scattered and exaggerated appearance of the human body in the avatar shape. These findings could enhance inter-avatar communication by considering brain activity.

## 1. Introduction

In recent years, human communication has expanded from face-to-face to communication via avatars in the metaverse as computerized agents that can be manipulated at will (Nowak and Fox, 2018). While communication in the real world takes place while observing the human body, avatar communication is characterized by the use of avatars that do not necessarily have a human-like appearance. Nevertheless, humans can communicate smoothly through avatars, but what mechanisms are involved in this process? When we understand that another person is human, we expect smooth communication; otherwise, the communication is expected to be more effortful (Timmerman et al., 2021). Therefore, recognition of the avatar’s humanity is important for smooth avatar-mediated communication. The appearance of humanness has two aspects: shape and motion. With avatars, we can imagine various shapes (human shape, angular shape, abbreviated shape, scattered shape, etc.) and motions (human motion, linear motion, etc.), with many degrees of freedom. The mirror neuron system (MNS) is a prominent brain phenomenon that has garnered significant attention in exploring the mechanisms underlying the perception of humanness. This MNS was initially found in a partial region of the ventral premotor cortex of the monkey (Gallese et al., 1996; Rizzolatti et al., 1996). In addition, the MNS was later found in the inferior parietal lobule (Fogassi et al., 2005). The MNS is a group of specialized neurons that are activated not only when an individual performs an action but also when they observe someone else performing that action. This intriguing neural mechanism has been proposed to play a crucial role in the comprehension of others’ actions and intentions (Fabbri-Destro and Rizzolatti, 2008). Various phenomena have been reported for the MNS, such as brain activity changes on electroencephalogram (EEG, μ-wave attenuation) and functional magnetic resonance imaging (fMRI) (Cross et al., 2016; Hortensius and Cross, 2018). Numerous reports on human-like shapes and motions showed MNS activation, and it is becoming clear that the MNS is activated in response to the appearance of humans. However, the influence of the perceived humanness of avatars on the MNS has not been clarified. When expanding the metaverse as a means of communication, it is important to elucidate the neural basis and apply this to future social implementation. To facilitate communication using avatars, it is necessary to investigate brain activities that support communication between humans using avatars.

MNS has been reported to respond to avatars with human-like shapes. Regarding the perception of the shape of an agent as a communication target, previous studies have compared human-shaped with non-human-shaped stimuli, such as animals or non-living objects. Specifically, it has been reported that ventral cortical visual tracts associated with the MNS were activated for human shapes while using extreme differences in depiction as the stimulus condition (Kriegeskorte et al., 2008; Wiggett et al., 2009). In the field of human perception, previous studies have also attempted to change cues to humanness by comparing humans with humans themselves and robots (Tong et al., 2000; Gobbini et al., 2011). Gobbini et al. showed similar involvement of central facial perceptual regions [spindle face area (FFA), occipital face area (OFA), and posterior superior temporal sulcus (pSTS)] when observing a human face and a human-like robot face (Gobbini et al., 2011). Furthermore, the neural core of face and body processing regions also corresponds to cartoons and schematic drawings of faces and bodies (Tong et al., 2000; Downing et al., 2001). Thus, the MNS including the cortical ventral visual pathways was activated based on physically human-like shapes’ avatars. In the MNS, one of the most important factors in the perception of humanness is that of a human-like shape.

Additionally, it was reported that both the human-like shape of the avatar and its human-like motions are related to the activation of the MNS. Previous studies have compared the effects of motion observations performed by humans and robots and found an involvement of sensorimotor brain regions associated with the MNS, collectively referred to as the action observation network (AON) which facilitated motion responses more when the agent was human than when it was not (Press, 2011; Gowen and Poliakoff, 2012). For example, observing human shape and motion increased motor priming, which is the effect of processing a primer as a preceding stimulus that promotes or inhibits the processing of a subsequent stimulus (Kilner et al., 2003; Press et al., 2006). Furthermore, the right premotor cortex was more involved during the observation of reaching motions performed by human hands than by robot hooks (Tai et al., 2004). These results regarding the avatar motions were consistent with an auto-similarity bias and more involvement of the MNS when observed agents have more humanness.

On the other hand, MNSs were also reported to be more broadly sensitive to avatar motions compared to avatar shapes. Previous studies on MNS activation in response to human motion suggested that the superior temporal sulcus associated with the MNS responds to human motion even in the absence of an explicit human shape (Allison et al., 2000; Blake and Shiffrar, 2007). Cross et al. (2012) reported that the premotor, parietal, and occipitotemporal regions respond more to robot-like motion than natural human motion. Similar response pattern for human and robot agents, suggesting a preference for robot-like motion is independent of the agent’s form. Findings indicated flexibility in response to novel actions by human and non-human agents. The MNS is sensitive to a broader range of action features beyond familiarity. Shimada (2010) used near-infrared spectroscopy to examine MNS activation in four conditions, a combination of shape (Human, Robot) and motion (Human, Robot). The results showed deactivation of the MNS when robot motion was combined with human shape. These suggest that a combination of shape and motion humanness of the avatar influences MNS activity, even though the avatar shape was not human-like.

Based on these prior reports, it is possible that avatar shape characteristics may alter MNS sensitivity when the avatar’s motions are not human-like. Exaggerated representation of body parts associated with humanity in avatar shape has the potential to be sensitive to MNS activation even if the avatar performs non-human-like motion. However, MNS activities for avatars in the virtual world, which have a high degree of freedom of shape and motion, have not been fully investigated or clarified, and this is a remaining problem. Specifically, avatars in a virtual environment can be configured to look and move even more freely than robots. Therefore, there is a need to investigate virtual avatars that resemble humans separately from robots. In addition, there has been no systematic investigation of the effect of the combination of shape and motion humanness on MNS activity from the viewpoint of EEG μ-wave attenuation.

**Figure 1:**
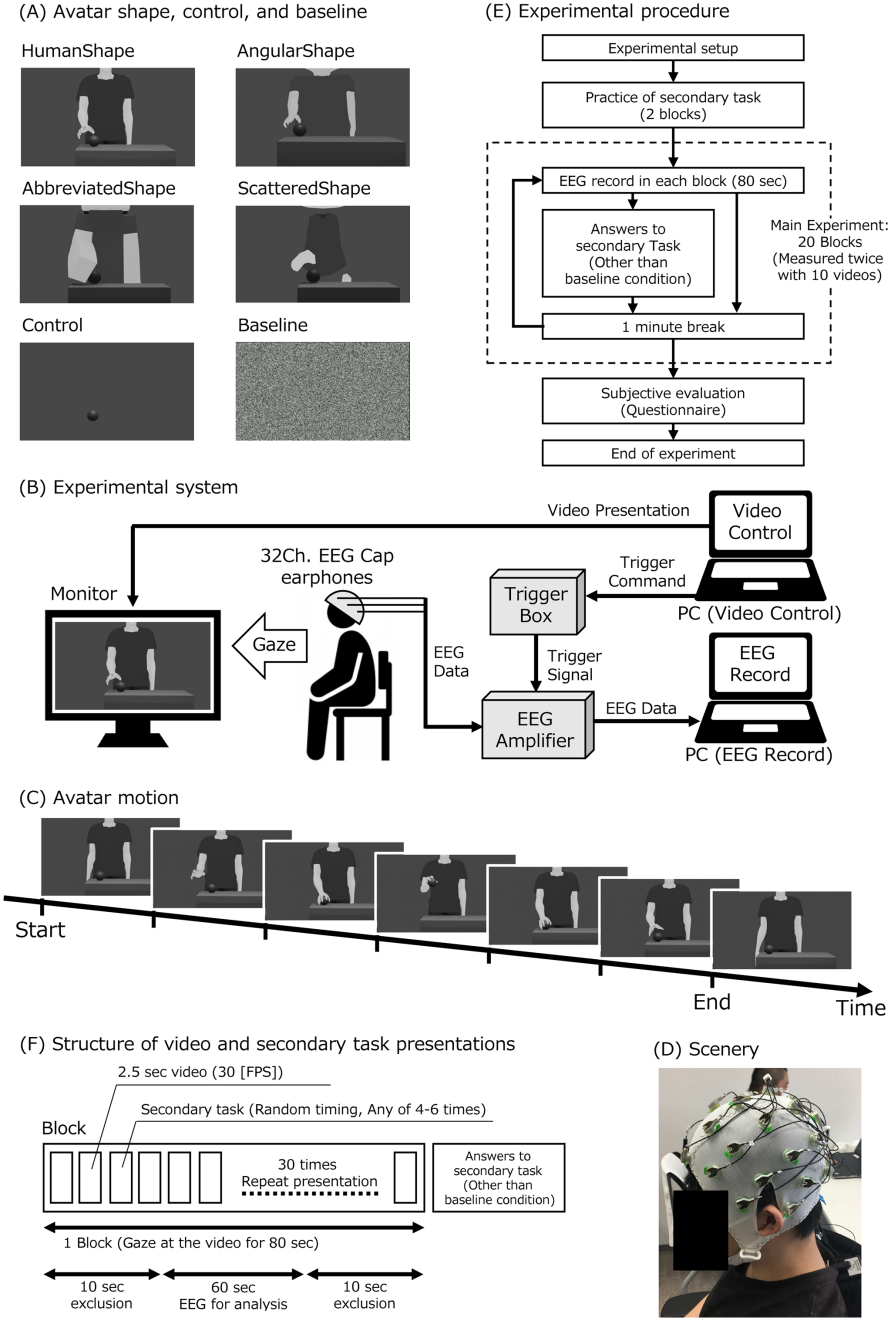
Experimental setup and protocol. **(A)** Videos for experimental conditions in avatar shapes, control, and baseline: there were six different videos for the experimental conditions. **(B)** Experimental setup: the participant observed the monitor displaying an avatar video. The video was presented from a PC for the video control (video-PC). The participant wore a 32-channel electroencephalogram (EEG) cap and earphones. The earphones provided a white noise sound. The EEG cap was connected to an EEG amplifier with a trigger box. The EEG data was recorded by a PC (measurement-PC) through the EEG amplifier. The video-PC sent triggers to a trigger box to synchronize the video presentation and the EEG data. The amplifier obtained the trigger signals from the trigger box and combined the trigger signal with the EEG data. **(C)** Avatar motion: the avatar movement was a grasping motion conducted with the hand. **(D)** Experimental scenery. **(E)** Experimental procedure: after preparing for the experiment, participants practiced the secondary task and then performed the main experiment. **(F)** Structure of video and secondary task presentations: one block consisted of 80 s of video observations. Each of the 10 different videos was shown twice. Thus, a total of 20 blocks were conducted. Each 80-s video consisted of 30 repetitions of a 2.5-s clip at 30 FPS. With random timing, four to six of the thirty 2.5-s videos were used as secondary tasks. After each block, participant responses to the secondary task were collected in all but the baseline condition.

Therefore, the purpose of this study was to investigate the effects of virtual avatars’ shape and motion humanness on MNS activity. This study measured and compared EEG μ-wave attenuation as a measure of MNS activity when observing avatars with human and nonhuman shapes and motions. This study used a combination of four avatar shape conditions and two avatar motion conditions to investigate whether there is a difference in MNS activation when participants observe two different motion patterns for each of the four different shapes of the avatar. The two different motion patterns of the avatar consisted of the HumanMotion and LinearMotion conditions for the human- and non-human-like motion patterns, respectively. Figure 1A illustrates the four different avatar shapes used in this experiment, which include HumanShape, AngularShape, AbbreviatedShape, and ScatteredShape for non-human-like shapes.

## 2. Materials and methods

### 2.1. Participants

Twenty-two healthy right-handed participants (9 males and 13 females) between the ages of 21 and 29 (mean 23.91 years, SD = 2.47) were tested. Data from 16 participants (8 males and 8 females) were ultimately used in the analysis [21–29 years old (mean 23.75 years old, SD = 2.17)]. All experimental subjects were confirmed to be neurotypical. Data from four participants were excluded from the analysis due to measurement problems (excessive eye blink artifacts) and from two participants who had problems performing the task. Participants were first briefed on precautions related to the experiment and signed a consent form. The study was approved by the Ethical Review Committee for Research Involving Human Subjects at the Tokyo Institute of Technology.

### 2.2. Experimental setup

The experimental setup is illustrated in Figure 1B. In the experiment, videos were played on a monitor in front of the participant, and EEG was recorded while the participants observed the videos. A PC for playing the videos (hereinafter: “video-PC”) was used to select and adjust the timing of the images played on the monitor, and the timing of the start and end of the image playback was sent as a trigger signal to the EEG which was recorded on a separate PC (hereinafter: “measurement-PC”).

### 2.3. Experimental equipment

A Dell Latitude 3,301 (OS: Windows 10 Pro 64-bit, CPU: Intel Core i5, memory: 8GB, Dell Technologies, Round Rock, TX) was used as the video-PC, and a dedicated MATLAB (MathWorks Inc., Natick, MA) script was created for video playback and trigger transmission. The videos were played on a 24-inch monitor (BenQ ET-0027-B; BenQ Corporation, Taipei, Taiwan). A 32-channel electroencephalograph actiCHamp (Brain Products GmbH, Gilching, Germany) was used for EEG measurements. The measured EEG was played back on the measurement-PC [Dell Precision 7,750 (OS: Windows 10 Pro 64-bit, CPU: Intel Core i7, memory: 64 GB), Dell Technologies] using Brain Vision Recorder (Brain Products GmbH). To record the trigger signals sent from MATLAB, a trigger box (National Instruments, Austin, TX) was used to connect the EEG to the video-PC.

### 2.4. Experimental conditions

Avatar shape and motion conditions were combined for the videos. There were four avatar shape conditions (HumanShape, AngularShape, AbbreviatedShape, and ScatteredShape) and two avatar motion conditions (HumanMotion and LinearMotion). Therefore, eight different videos were created for the conditions in which the avatars would appear: HumanShape & HumanMotion, HumanShape & LinearMotion, AngularShape & HumanMotion, AngularShape & LinearMotion, AbbreviatedShape & HumanMotion, AbbreviatedShape & LinearMotion, ScatteredShape & HumanMotion, and ScatteredShape & LinearMotion.

In the avatar motion conditions, as shown in Figure 1C, the motion performed by the avatar was to reach for the ball on the desk, pick it up, and place it on the desk again. It was created so that there would be no discomfort at the joints of the animation even after repeated playback. In the HumanMotion condition, as an animation that reproduced the curvilinear trajectory and speed distribution characteristics of human motion, we used the CG creation software Blender (Blender Institute, Amsterdam, Netherlands) to create an animation of the actual motion of grabbing the ball on the desk, based on previous research (Cross et al., 2012). The motion of the HumanMotion condition was traced frame by frame using Blender.

To create a video of an avatar performing a human-like ball grasping motion for the HumanMotion condition, the study first recorded a person grasping and lifting a desk ball before placing it back on the desk using a video camera. The video was then analyzed frame-by-frame, with the position of each joint in the person’s body recorded for each frame. Using this data, this study determined the position of each joint in the avatar’s body for each frame in its video. Motion tracing techniques were then employed to generate a video of the avatar performing the same motion as the person, resulting in a realistic simulation of the ball’s grasping motion.

In the LinearMotion condition, non-human, linear, and constant velocity animations were created based on previous research (Stadler et al., 2012) by tracing the posture from the video using only the major points in the motion as keyframes and using linear frame interpolation for the motion between keyframes. To create the LinearMotion condition, two types of information were utilized: joint position data from video recordings of a human body, and continuous position data to connect each joint position linearly. The joint position data was used to time the seven postures in the ball grasping motion, as illustrated in Figure 1C. These timings included the initial posture, full hand lift, ball grasping, full ball lift, ball placement, full hand lifts away from the ball, and final posture. The position of each joint for these timings was determined using the joint position data from the recoded actual human video, just as in the HumanMotion condition. The linear completion function was then used to generate the linear movement between each joint position for each of the seven timings. This resulted in non-biological motion, as the movement to the hand position set in each keyframe was animated at a constant velocity and linear.

In the avatar shape conditions, as shown in Figure 1A, the following four types of avatar shapes were prepared. It was anticipated that the HumanShape condition, which has body proportions and curvilinear contours similar to those of a real human being, would be perceived as highly human-like. The AngularShape condition, which was deformed to have an unrealistic body shape, was expected to be rated lower than the human avatar and higher than the other two types of avatars in terms of human-like characteristics. The AbbreviatedShape and ScatteredShape conditions, which were designed to create a significantly unrealistic shape for a human-like avatar, were assumed to have a low human-like rating. The AbbreviatedShape condition represented an avatar shape with the details of the shape of the HumanShape condition omitted. The ScatteredShape condition represented an avatar shape that omitted the continuity of the HumanShape condition.

In addition, videos for the control and baseline conditions were created. For the control condition, a video with a hidden avatar was created that only showed the ball movement. In this video, the MNS was expected to be inactive because no humanness information was present in shape and motion. Videos for the baseline condition, which were needed for the EEG analysis, were created using white noise. Several Gaussian noise images with a standard deviation of 100 were generated on Google Colaboratory (Google, Mountain View, CA). The frame rate for all videos was 30 fps, and the length was approximately 2.5 s.

In summary, the participants in the experiment watched a total of 10 types of videos: four conditions for avatar shape times two conditions for the motion assigned to each avatar plus videos from the control and baseline conditions.

The ten videos for each experimental condition used in the study can be viewed in the Supplementary material.

### 2.5. Experimental protocol

As shown in Figures 1B,D, participants sat on a chair, wearing an electrode cap for EEG measurement and earphones, and observed videos played on a monitor placed approximately 90 cm in front of them. The participants were instructed to place their chins on a chin rest fixed to a desk to reduce head movements that could cause noise in the EEG data. By adjusting the position and height of the chair, the participants were able to observe the monitor in front of them in a comfortable position.

The videos were presented on the playback monitor with a height of approximately 8 cm and a width of 13.3 cm. To block external sounds, white noise was played through the earphones while the videos were being played. Immediately before the start of the video playback, the participants were instructed to observe the video carefully and to avoid body movements and blinking of the eyes as much as possible.

As shown in Figure 1E, the EEG was measured twice in each condition for a total of 20 times. In each measurement block, only one type of video (approximately 80 s) was presented. After a block of measurements was completed, there was a break of at least one minute before the next block started. As practice for the secondary task, two blocks were presented before the 20 blocks of EEG measurements, using videos of scenery for testing. If there were difficulties understanding the content of the secondary task, the number of practice blocks was added as needed, and the EEG measurement block was started when the participants were able to engage in the task without problems. In the 20 EEG measurement blocks, the videos for each condition were presented in random order for the first 10 blocks, and the order of the first half was repeated for the second 10 blocks. The random order of blocks for each condition was changed with counterbalancing for each subject. This was to avoid generalization effects for this experiment.

As shown in Figure 1F, the experiment included approximately 80 s of video with 30 repetitions of approximately 2.5 s of video for each condition. Referring to previous studies (Oberman et al., 2005), a secondary task was performed in all conditions, except the baseline condition, to focus attention on the video. In the secondary task, the repeated playback of the video stopped four to six times during the full video, and participants were asked to silently count the number of stop times without using their fingers and respond verbally after the video ended. Participants who answered the task correctly less than 50% of the time were considered to have failed to pay sufficient attention to the performed experiment and were excluded from the data analysis. Data from two participants who had problems performing the task were excluded from the analysis.

### 2.6. Data collection: EEG

During the experiment, participants wore dedicated 32-channel electrode caps (EASYCAP GmbH, Woerthsee, Germany) on their heads. Measurements were taken at a sampling rate of 500 Hz, while the impedance between each electrode and the scalp was kept below 10 kΩ by applying a dedicated gel (SuperVisc Gel, EASYCAP GmbH). Fz was used as the reference electrode for EEG recording. The ground electrode was placed on the forehead (near FPz).

### 2.7. Data analysis: EEG

EEGLAB (Swartz Center for Computational Neuroscience, San Diego, CA), a MATLAB toolbox, was used to analyze the EEG data. After removing power line noise from the data of each electrode, a bandpass filter from 0.1 to 30 Hz was applied, and the data were re-referenced to TP9 and TP10 as both mastoids. Each experimental condition’s video was presented in a randomized order for every block, as illustrated in Figure 1F. During each block, participants viewed the video for a specific experimental condition 30 times consecutively, with each block lasting 80 s. The first 10 s of data and the last 10 s of data for each block were excluded from the analysis to eliminate the effect of attention due to the start and end of the video playback. EEG data was recorded for 80 s during each block. After excluding the first and last 10 s of data, 60 s of data were left for analysis. The data was segmented into epochs of 1,024 samples (approximately every 2 s), and frequency analysis was performed on each epoch using the fast Fourier transform (FFT). The power of the μ-waves (oscillations in the 8–13 Hz band) in each segment was determined using FFT after excluding segments containing strong artifacts caused by eye blinks or body movements.

The average power of the μ-waves in each condition was divided by the average power of the μ-waves in the baseline condition, and the log-transformed value was used as the amount of change in the μ-waves for the evaluation. By using the ratio to the baseline condition, comparisons can be made between conditions, while minimizing the effect of interindividual differences. A negative log ratio means that the μ-waves were significantly reduced compared to the baseline condition, indicating μ-wave attenuation and suggesting MNS activation. Student’s *t*-test was performed on the log ratios of the μ-waves in each condition to determine whether they were significantly negative and different from zero. Electrodes covering the central sulcus areas on either side of the sulcus (C3 and C4), which are considered to reflect the activity of the MNS, were the target of the analysis.

### 2.8. Questionnaire

The avatar shape was subjectively evaluated by displaying one still image of each avatar on a video playback monitor. Participants responded orally to a set of questions about the humanness of the avatar shape, which were based on components from the uncanny valley study (Kätsyri et al., 2017). The questionnaire consisted of three items: human-like, exaggerated, and cartoonish. Participants rated each item on a 7-point scale ranging from strongly agree (+3) to strongly disagree (−3), with a score of 0 indicating neutrality. To avoid response bias due to order, the avatars and questions were randomly presented. The subjective evaluation was conducted at the end of each participant’s experiment. The participant’s responses to the three items were quantified using the humanlike-scale, the exaggerated-scale, and the cartoonish-scale, and the average score of the three items was calculated as the humanness-score. It is important to note that when computing the average, the sign of the humanlike-scale was unchanged, but the signs of the exaggerated-scale and the cartoonish-scale were reversed (i.e., positive values become negative and vice versa).

## 3. Results

**Figure 2:**
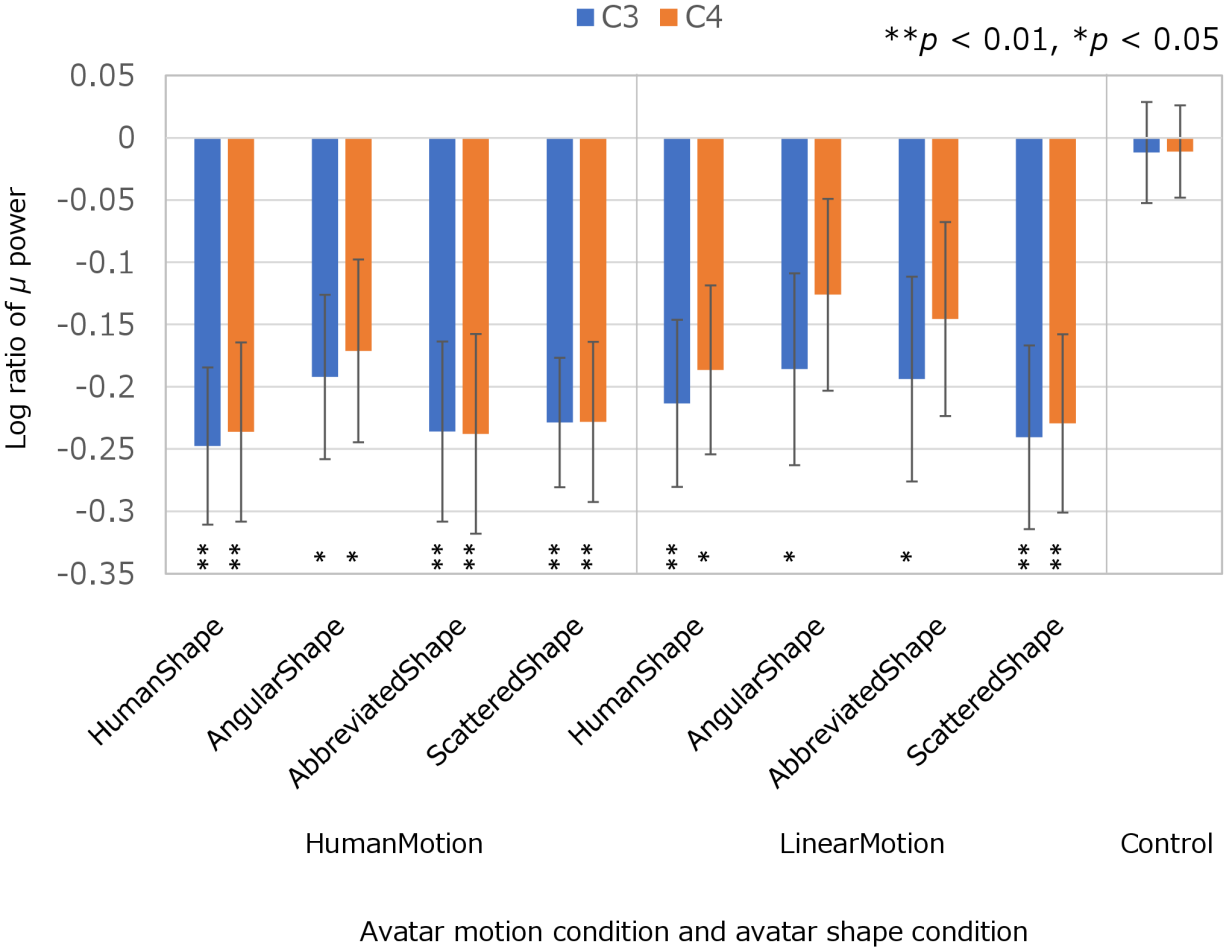
Log ratios of μ power in C3 and C4 electroencephalogram channels in avatar motion and avatar shape conditions. The log ratio of μ power is the log ratio of the μ power in each condition to the μ power in the baseline condition, in C3 and C4, respectively. A negative value for the Log ratio of μ power means that the μ-wave was significantly reduced compared to the baseline condition, indicating μ-wave attenuation. To confirm μ-wave attenuation, Student’s *t*-test was performed on the Log ratios of the μ-waves in each condition. “**” (*p* < 0.01) and “*” (*p* < 0.05) denote statistical significance.

The experimental conditions were compared based on the results of μ-wave attenuation in the C3 and C4 channels of the EEG which are shown in Figure 2; Table 1. The Kolmogorov–Smirnov test for normality confirmed that the values obtained under all conditions followed a standard normal distribution (*p* < 0.05 for both C3 and C4 channels in all conditions). Student’s *t*-test was performed to determine whether the μ-wave attenuation in each condition was significant, meaning that the log ratio was significantly negative. In the control condition, the μ-waves were not significantly attenuated in either the C3 or C4 channels. In the HumanMotion condition, significant μ-wave attenuation was observed in both the C3 and C4 channels, regardless of the avatar shape. On the other hand, the LinearMotion condition, in which non-biological motion was applied, showed different trends among conditions. Significant μ-wave attenuation was observed in both the C3 and C4 channels for the HumanShape and ScatteredShape conditions, whereas it was only observed in the C3 channel for the AngularShape and AbbreviatedShape conditions.

**Table 1:** Log ratios of μ power in C3 and C4 electroencephalogram channels in avatar motion and avatar shape conditions.

Avatar motion condition	Avatar shape condition	C3	C4
HumanMotion	HumanShape	−0.247 (0.063)**	−0.236 (0.072)**
	AngularShape	−0.192 (0.066)*	−0.171 (0.073)*
	AbbreviatedShape	−0.236 (0.072)**	−0.238 (0.080)**
	ScatteredShape	−0.229 (0.052)**	−0.228 (0.064)**
LinearMotion	HumanShape	−0.213 (0.067)**	−0.186 (0.068)*
	AngularShape	−0.186 (0.077)*	−0.126 (0.077)
	AbbreviatedShape	−0.194 (0.082)*	−0.146 (0.078)
	ScatteredShape	−0.240 (0.074)**	−0.229 (0.072)**
Control		−0.012 (0.041)	−0.011 (0.037)

**p < 0.01, *p < 0.05. The log ratio of μ power is the log ratio of the μ power in each condition to the μ power in the baseline condition, on C3 and C4, respectively. “**” (*p* < 0.01) and “*” (*p* < 0.05) denote statistical significance.

Additionally, this study required participants to observe a video about the avatars. Therefore, a secondary task was conducted to ascertain whether or not participants had observed them. In the secondary task, the video was stopped multiple times at random, and participants were asked to silently count the number of stops without using their fingers and to respond verbally after the video ended. Participants who answered less than 50% of the tasks correctly were considered to have failed to pay sufficient attention to the experiment being conducted and were excluded from the data analysis. As a supplementary result, the average percentage of correct responses to the secondary task was 90.28% (SD = 6.94%). The total number of incorrect responses for the 16 participants in each condition is listed in Table 2.

**Table 2:** Number of wrong answers in the secondary task per condition.

Avatar motion condition	Avatar shape condition	Number of wrong answers
HumanMotion	HumanShape	2
	AngularShape	3
	AbbreviatedShape	2
	ScatteredShape	3
LinearMotion	HumanShape	5
	AngularShape	3
	AbbreviatedShape	5
	ScatteredShape	2
Control		2

The study utilized a questionnaire to subjectively evaluate the humanness of the avatar using three indices from the uncanny valley study (Kätsyri et al., 2017): humanlike-scale [7 levels: strong positive (+3), do not know/neither (0), strong negative (−3)], exaggerated-scale [7 levels: strong positive (+3), do not know/neither (0), strong negative (−3)], and cartoonish-scale [7 levels: strong positive (+3), do not know/neither (0), strong negative (−3)]. The questionnaire was used to obtain responses to these indices, and the average of the responses was calculated as the humanness-score. It should be noted that when calculating the average, the sign of the humanlike-scale was unchanged, but the signs of the numbers for the exaggerated-scale and the cartoonish-scale were reversed. One sample Wilcoxon signed-rank test, a non-parametric test whose location parameter under the null hypothesis is zero, was used to analyze whether there were any differences in subjective evaluation results for the four avatar shape conditions: HumanShape, AbbreviatedShape, ScatteredShape, and AngularShape. The statistical analysis yielded the following humanness score (mean ± standard deviation) and *p*-values for each avatar shape condition: HumanShape (2.0 ± 0.8, ***p < 0.001), AbbreviatedShape (−1.9 ± 0.9, ***p < 0.001), ScatteredShape (−1.6 ± 1.0, *p* < ***0.001), and AngularShape (0.5 ± 1.3, *p* = 0.2). These findings suggest that the HumanShape condition provided human-like avatar shape information to the experimental participants, whereas the AbbreviatedShape and the ScatteredShape conditions provided non-human-like avatar shape information.

## 4. Discussion

Using the scales of humanness (Kätsyri et al., 2017), the subjective evaluation of the humanness of the avatar shape was assessed in the current study. The humanness score, which was the average of the responses to the three questions, indicated that the HumanShape condition had significantly more humanness. Conversely, the AbbreviatedShape and ScatteredShape conditions had relatively unrealistic human shapes. The humanness score of the AngularShape condition could not be determined whether it was human-like or non-human-like. Unlike previous studies where the differences in the appearance and humanness of agents were expressed in terms of humans and robots, and the differences were clear (Shimada, 2010; Cross et al., 2012). In the current study, various appearances of humanness were set up by changing the appearance of the avatars in a step-by-step fashion. It was assumed that there would be a mixture of human-like and non-human-like shapes. Indeed, these subjective evaluation results were consistent with the assumptions of this current study, suggesting that the experimental conditions were set appropriately. Therefore, this study could discuss the EEG analyses in human-like avatars with the HumanShape condition and in non-human-like avatars with the AbbreviatedShape and ScatteredShape conditions. However, the EEG analysis with the AngularShape condition could not be discussed because it was difficult to determine whether the avatar shape was human-like or non-human-like based on the result of analyzing the questionnaire.

This second discussion is with respect to the results of EEG analysis for each avatar experimental condition as objective evaluation. In this study, μ-wave attenuation at electrodes covering the central sulcus areas on either side of the sulcus (C3 and C4), which reflect activity in the MNS, was used as an objective evaluation index of brain activity. For each experimental condition, the value of the log ratio of the EEG power between the μ-wave band and baseline condition was analyzed to determine whether significant μ-wave attenuation was present. First, no significant μ-wave attenuation was observed in the control condition, in which only a ball motion was presented. This result replicated previous studies (Oberman et al., 2005).

In the HumanMotion condition with biological motion, significant μ-wave attenuation was observed in channels C3 and C4 in all four avatar appearance conditions, which means that brain activity based on changes in avatar shape suggested similar trends. On the other hand, in the LinearMotion condition, in which non-biological motion was added, there were differences in activity trends depending on the avatar shape. Significant μ-wave attenuation was observed in channels C3 and C4 for the HumanShape and ScatteredShape conditions. In contrast, the AngularShape and AbbreviatedShape conditions only showed significant μ-wave attenuation in C3 but not in C4.

The HumanShape condition showed relatively human-like shapes compared to the AbbreviatedShape and ScatteredShape conditions. In the HumanMotion condition, the MNS was activated when the participants observed avatars consisting of a combination of the HumanMotion condition and any avatar shape conditions, meaning that the observation of human-like motion was associated with MNS activity. On the other hand, in the LinearMotion condition, MNS activation varied according to the avatar shape conditions.

The AbbreviatedShape and ScatteredShape conditions, which appeared relatively less human-like, also showed different results. This is a novel piece of evidence that demonstrates that even if humans do not consciously perceive the avatar as human-like, they can still perceive it to be human-like.

In interpreting the experimental results of this study, it is important to note that a previous study by Oberman et al. (2007) investigated μ-wave attenuation at electrodes covering the central sulcus areas on either side of the sulcus (C3 and C4) when viewing a human hand and a robot hand. The results showed that μ-wave attenuation was significant in channels C3 and C4 when looking at the human hand, indicating that the MNS was activated. However, when viewing the robot hand, μ-wave attenuation was only significant in C3 but not in C4, indicating that the MNS was not activated. Their study examined the correspondence between channels C3 and C4 and significant μ-wave attenuation in detail. They report that when μ-wave attenuation occurs in both the C3 and C4 channels, the MNS is activated, whereas when μ-wave attenuation only occurs in C3 but not in C4, the MNS is not activated.

In the HumanShape condition, μ-wave attenuation was observed in the C3 and C4 channels in both HumanMotion and LinearMotion conditions. Thus, activation of the MNS was observed for human-like avatars. Similarly, in previous studies, the ventral cortical visual pathways associated with the MNS were activated in response to human-like depictions, compared to non-human stimuli such as animals or non-living things (Kriegeskorte et al., 2008; Wiggett et al., 2009).

In the HumanMotion condition, μ-wave attenuation was observed in both C3 and C4 for all shapes: HumanShape, AbbreviatedShape, and ScatteredShape. Thus, activation of the MNS was observed for avatar motion humanness. Similarly, in previous studies, the superior temporal sulcus associated with the MNS responded to human-like actions, even in the absence of a clear human form (Allison et al., 2000; Blake and Shiffrar, 2007). When the agent was human, the responses in the MNS-related brain regions were enhanced compared to when the agent was not human (Kilner et al., 2003; Press et al., 2006; Press, 2011).

With respect to avatar motion without humanness, the results varied with avatar shape conditions. For the ScatteredShape with the LinearMotion condition, μ-wave attenuation was observed in both C3 and C4, indicating activation of the MNS. In contrast, for the AbbreviatedShape condition with the LinearMotion, μ-wave attenuation was observed only in C3 but not in C4, indicating non-activation of the MNS. Thus, in the LinearMotion condition, when the avatar shape was varied and human-like and non-human-like motions were performed, the MNS showed different activation patterns for each condition. This indicated that it is important to take their characteristics into account to facilitate communication by using avatars.

In the ScatteredShape condition, MNS activation was observed for both the HumanMotion and LinearMotion conditions. The avatar in the ScatteredShape condition was not human-like. In other words, in avatars that were non-human-like, the MNS was activated when non-human-like motions were performed.

Similar results have been reported in previous studies. When participants saw actions performed by humans or a robot, no differences in brain responses were observed (Gazzola et al., 2007). The left frontoparietal sulcus, associated with the MNS, responded similarly when participants observed geometric shapes and when humans performed target-oriented actions (Ramsey and Hamilton, 2010). In comparison to human visual cues, the perception of non-humans elicited even greater responses in the MNS-related brain regions (Cross et al., 2012; Saygin and Stadler, 2012). Compared to natural free-flowing dance movements that are consistent with the human motor repertoire, the MNS showed a large involvement when observing difficult robotic movements (Cross et al., 2012). The MNS was more sensitive to androids, or robots dressed as humans, compared to real-life humans or robot actors (Saygin and Stadler, 2012). Thus, our results showing sensitivity to an avatar with a combination of non-human-like shapes and motions are consistent with those of previous studies.

In the AbbreviatedShape condition, MNS activation was observed in the HumanMotion, but not in the LinearMotion condition. The MNS was not activated when the avatar, which was non-human-like in appearance, performed non-human-like actions.

In previous studies, the MNS associated with the right premotor cortex was more involved during the observation of reaching movements performed by human hands compared to robot crimpers (Tai et al., 2004). Thus, our results are consistent with those of previous studies that showed that the MNS was not activated for the combination of non-human-like shapes and motions. Moreover, the results of the comparison between the LinearMotion condition with the ScatteredShape condition and the LinearMotion condition with the AbbreviatedShape condition indicate that the MNS was activated in some cases, even when non-human-like shapes and movements were combined. These results, previously reported separately in different studies, were observed for the first time simultaneously under common experimental conditions. The essential humanness difference between the ScatteredShape and AbbreviatedShape conditions was whether the avatar shape was depicted continuously or discontinuously. This visual perception of the structure of human details may be an important factor in the divergence. In other words, the discontinuous shape of the avatar body has the potential to be perceived by the MNS as an essential element that keeps it away from humanness, resulting in a more sensitive response of the MNS to objects that are not human-like.

As a result of the current research, even if the avatar shapes of the AbbreviatedShape and the ScatteredShape condition were perceived as non-human-like, the MNS responded to the ScatteredShape in the LinearMotion condition, but not to the AbbreviatedShape condition. This suggests that there is a condition for the shape to which the MNS is sensitive when we consider an avatar with a less human-like shape. The main difference between the AbbreviatedShape and the ScatteredShape condition was whether the avatar’s body parts were drawn continuously or discontinuously. Therefore, when an avatar’s movements are not human-like, the MNS may perceive the continuous nature of the shape as dehumanizing. It has been suggested that if the avatar’s movements were not human-like, the MNS would be sensitive to the discontinuity of the avatar’s shape. Shimada (2010) reported that deactivation of the MNS was not observed when a robot-motion was executed by a robot-shape. Such robotic shapes consisted of small rigid parts connected together and were characterized by convex, uneven, jagged, and discontinuous contours. In addition, the avatar’s movement was a grasping motion executed by its hands. Cross et al. (2012) reported that a large involvement of the MNS was shown when observing stiff robotic motions performed by the human body. In this study, the ScatteredShape condition avatar placed a greater emphasis on the functional part of the hand than the AbbreviatedShape condition, as the position of the hand was clearly visible. This suggests that the MNS may be particularly sensitive to functional body parts, such as a hand grasping a ball, when the deformed human-shaped avatar, which looks less human-like, is accompanied by linear robot-like movements.

In this study, the videos used in each experimental condition did not depict the avatar’s face, which prevented any MNS effects related to facial depiction. Previous research by Gobbini et al. (2011) demonstrated that the central facial perceptual regions (FFA, OFA, and pSTS) exhibit similar involvement when observing a human face and a human-like robot face, suggesting that the cortical ventral visual pathways associated with the MNS are based on physically human-like shapes rather than actual faces and bodies. Moreover, previous studies have suggested that the neural core of face and body processing regions also corresponds to cartoons and schematic drawings of faces and bodies (Tong et al., 2000; Downing et al., 2001). Based on these predictions, it is possible that adding additional facial information to the avatar’s appearance in the video used in the experimental conditions of this study may not significantly affect MNS activation.

An interesting experimental result of this study is that the exaggerated shape of the avatar’s arms may have affected the activation of the MNS when the avatar’s movements were not human-like. Indeed, such results were obtained under the experimental conditions of the ScatteredShape avatar. This may suggest that exaggerated human functional body parts may be specifically related to the perception of humanness. Calvo-Merino et al. (2006) investigated whether the mirror system in the human brain relies on specialized motor representations or general visual inference and knowledge processes to understand actions. Using fMRI, the researchers examined motor influences on action observation in expert dancers. They found that mirror circuits generate a purely motor response beyond visual representations of action. This suggests that actions are understood both visually and motorically. Saygin et al. (2004) examined the impact of motion cues on object and event perception, focusing on point-light biological motion. Using fMRI, the researchers found that these animations activate frontal cortex areas associated with action observation. The study suggests that even simplified displays like point-light animations engage the frontal cortex areas, with the observer’s motor system contributing to filling in the information conveyed by motion cues. In the current study, the MNS was activated for the avatar’s human-like movements, consistent with this finding. On the other hand, for non-human-like movements, the MNS was considered to be activated not only when the avatar was human-like, but also when the avatar’s hand part had an exaggerated shape. This observation indicates that the MNS might be involved in responding to body parts that have a potential impact on the external world, such as the human hand and arm.

This study used EEG measurements to detect MNS activation. However, recent studies have attempted to use more detailed observation of brain activity, such as fMRI (Cross et al., 2016). The combined use of such techniques is expected to reveal more detailed characteristics of brain activity and co-occurrence relationships among brain regions. Additionally, in communication within cyberspace, including the metaverse, the MNS may be sensitive and attentive to body parts directly related to the avatar’s movements. Therefore, by adjusting the shape and movement characteristics of avatars, taking into account the MNS and other features of brain activity, communication in the metaverse has the potential to be facilitated and made more efficient, supporting the formation of human interactions.

The limitations of this study are as follows. The experiments were conducted with four avatar shape conditions and two avatar motion conditions. Further details would require setting up additional experimental conditions and conducting additional experiments. Appearances such as the color of all avatars in this study were similar. However, there is a variety of appearances when actual communication is assumed. Therefore, further investigations of the effects of varied avatar appearances are required. EEG μ-wave attenuation was used as an objective evaluation index. However, previous studies have pointed out that mu attenuation may be disrupted by changes in attention because the mu frequency band overlaps with the alpha frequency band, which is sensitive to changes in attention (Hobson and Bishop, 2016; Hobson and Bishop, 2017). In addition, the spatial resolution of EEG is worse than that of fMRI. Therefore, fMRI or other high-resolution measurement methods may be useful to further clarify our results.

In the AngularShape condition, subjective evaluation using a questionnaire did not provide a clear distinction between whether the avatar of the AngularShape condition was perceived as human-like or non-human-like. Therefore, the finer condition differences included in the AngularShape condition were not considered. Investigating the details of humanness is a topic that requires further exploration in future studies.

This study builds upon previous research by Oberman et al. (2007) and presents the results of experiments conducted using avatars with different characteristics. In this previous study, data from 17 experimental participants were used for analysis. The analysis in the current study utilized data from 16 experimental participants. However, to fully understand the relationship between these avatar characteristics and the MNS, further inter-condition comparisons using statistical methods are needed, which were not conducted in the previous study. The current study, based on the experimental design of the previous study, did not perform comparisons between conditions using statistical analyses such as analysis of variance (ANOVA). For reference, Supplementary materials for this aspect as a first step is shown in Result S1. As a result, this study was unable to fully discuss differences between conditions, representing one of the study’s limitations. To address this limitation, future studies should include more experimental participants to conduct necessary comparisons to clarify this relationship.

This study designed an experiment based on the previous study by Oberman et al. (2007) and performed EEG analysis mainly focusing on the electrode channels of C3 and C4. However, other EEG channels, particularly the CP and P electrode channels, may also be relevant. Therefore, further analysis of these electrode channels will be required in future experiments.

In this study, to record baseline activity, a separate baseline condition was used consisting of white noise. On the other hand, in previous reports (Hobson and Bishop, 2016), the superiority of within-trial baselines has clearly been shown. Therefore, the introduction of such a within-trial baseline may be considered in further experiments in the future.

The main manipulation in this study involved the presence/absence of human-like motion and shape in the avatar. However, it is known that the observation of goal-directed actions activates the MNS regardless of the specific motion and shape of the avatar (Pelphrey et al., 2003; Ramsey and Hamilton, 2010; Shimada, 2010). The task in the current study involved a reaching-grasping goal-directed action, which was composed of several motor acts that were goal-oriented. On the contrary, a non-goal-oriented movement would be a simple intransitive movement (e.g., random movements of the upper limb). In this way, it is possible that this kind of influence of goal-directed actions could not be completely eliminated in this study, and examination of this aspect is considered to be a limitation of this study and a future issue.

To date, no previous studies have investigated the EEG-based MNS while simultaneously manipulating both human-like appearance and movement. To the best of our knowledge, this is the first experimental investigation to explore this perspective, providing initial insight into the characteristics of EEG-based MNS when simultaneously altering human-like appearance and movement. Furthermore, this study addresses the issue of inconsistency in previous research regarding the combination of human form and movement, which has been suggested to influence MNS activity. However, while this study contributes to our understanding of MNS activation, the results did not fully confirm the consistency of previous research, and further investigation is necessary to fully elucidate the complex relationship between appearance and movement in the context of MNS activity.

Regarding the analyzed regions of this experiment, in addition to the parietal cortex, the premotor cortex (both dorsal and ventral sectors) has been reported to be involved in the processing of action goals and kinematics during the observation of robots and human agents (Pelphrey et al., 2003; Ramsey and Hamilton, 2010; Shimada, 2010). However, there is no further data or analysis related to these aspects in this study, which is needed in future work. Thus, to further investigate the subject at hand, it is essential to explore potential variations in the participants’ task, particularly by instructing them to observe the avatar with the intention of imitating its actions. This variation prompts the question of what type of avatar should be utilized for such investigations. It can be hypothesized that when participants are required to encode the action goal and subsequently transform this information into a motor output, the engagement of the MNS may differ. Such experimental verification will be required in the future.

## 5. Conclusion

This study aimed to explore the influence of avatar shape and motion humanness on MNS activity using EEG μ-wave attenuation. Four avatar shapes were used (HumanShape, AngularShape, AbbreviatedShape, and ScatteredShape) and two motions were examined (HumanMotion and LinearMotion). Results from a questionnaire revealed that HumanMotion was perceived as human-like, while AbbreviatedShape and ScatteredShape were seen as non-human-like. Here, AngularShape’s humanity was indefinite. As expected, the MNS was activated for avatars with human-like shapes and/or motion. However, for non-human-like motion, there were differences in activity trends depending on the avatar shape. Specifically, avatars with HumanShape and ScatteredShape in LinearMotion activated the MNS, while the MNS was indifferent to avatars with AngularShape and AbbreviatedShape in LinearMotion. Comparing the ScatteredShape condition avatar with the AbbreviatedShape condition, the ScatteredShape condition avatar placed a greater emphasis on the functional part of the hand. The position of the hand was clearly visible, indicating that the MNS may be particularly sensitive to functional body parts, such as a hand grasping a ball, even when the deformed human-shaped avatar looks less human-like and has robot-like movements. These findings suggest that when avatars make non-human-like motions, the MNS is activated not only for human-like appearance but also for the scattered and exaggerated appearance of the human body in the avatar shape. Overall, this study contributes to research on inter-avatar communication, considering brain activity and the MNS, and has the potential to facilitate interpersonal interactions in cyberspace, including the metaverse with highly flexible avatars.

## Data availability statement

The raw data supporting the conclusions of this article will be made available by the authors, without undue reservation.

## Ethics statement

The studies involving humans were approved by the Ethical Review Committee for Research Involving Human Subjects at the Tokyo Institute of Technology. The studies were conducted in accordance with the local legislation and institutional requirements. The participants provided their written informed consent to participate in this study.

## Author contributions

YuM, HU, and YoM contributed to the conception and design of the study, developed and implemented the system, conducted the evaluation experiment, discussed and interpreted the results, drafted the manuscript, and reviewed the manuscript. All authors contributed to the article and approved the submitted version.

## Supplementary material

SUPPLEMENTARY MATERIALS Video 1: Control, Video 2: Abbreviated Shape & Human Motion, Video 3: Abbreviated Shape & Linear Motion, Video 4: Angular Shape & Human Motion, Video 5: Angular Shape & Linear Motion, Video 6: Human Shape & Human Motion, Video 7: Human Shape & Linear Motion, Video 8: Scattered Shape & Human Motion, Video 9: Scattered Shape & Linear Motion, and Video 10: Baseline.
