# Supplementary material for: Effects of avatar shape and motion on mirror neuron system activity
Source: Front Hum Neurosci. 2023 Oct 4;17:1173185. doi: 10.3389/fnhum.2023.1173185 (PMC10582709; doi:10.3389/fnhum.2023.1173185)
Supplement: SUPPLEMENTARY MATERIALS — Video 1: Control, Video 2: Abbreviated Shape & Human Motion, Video 3: Abbreviated Shape & Linear Motion, Video 4: Angular Shape & Human Motion, Video 5: Angular Shape & Linear Motion, Video 6: Human Shape & Human Motion, Video 7: Human Shape & Linear Motion, Video 8: Scattered Shape & Human Motion, Video 9: Scattered Shape & Linear Motion, and Video 10: Baseline. [file Data_Sheet_1.pdf]

Effects of Avatar Shape and Motion on Mirror Neuron System Activity

Yuki Miyamoto[1], Hirotaka Uchitomi[2*], Yoshihiro Miyake[2]

[1]Department of Systems and Control Engineering, School of Engineering, Tokyo Institute of Technology, Yokohama, 226-8502, Japan

[2]Department of Computer Science, School of Computing, Tokyo Institute of Technology, Yokohama, 226-8502, Japan

*** Correspondence:**
Hirotaka Uchitomi
uchitomi@c.titech.ac.jp

Keywords: humanness, avatar, mirror neuron system, μ-wave attenuation, electroencephalogram

Supplementary materials

Result S1. Statistical results for evaluation indexes in Avatar Motion and Avatar Shape Conditions. "*" indicates a significant difference with $p < 0.05$, "***" indicates a significant difference with $p < 0.001$, and "n.s." indicates a non-significant difference in the statistical evaluation results.

The analysis of variance (ANOVA) for the log ratios of μ power in C3 electroencephalogram channels in both avatar motion and avatar shape conditions was conducted. The results showed that the main effect of the avatar shape did not yield a significant difference ($F(3, 45) = 0.492$, $p = 0.7$, n.s.). Similarly, the main effect of the avatar motion did not show a significant difference ($F(1, 15) = 0.388$, $p = 0.5$, n.s.). The interaction effect between avatar shape and avatar motion also did not reveal a significant difference ($F(3, 45) = 0.592$, $p = 0.6$, n.s.).

For the ANOVA of the log ratios of μ power in C4 electroencephalogram channels in avatar motion and avatar shape conditions, the results indicated that the main effect of the avatar shape was not statistically significant ($F(3, 45) = 1.270$, $p = 0.3$, n.s.). Conversely, the main effect of the avatar motion did show a significant difference, with log ratios of μ power in HumanMotion being significantly lower than those in LinearMotion in C4 electroencephalogram channels ($F(1, 15) = 5.675$, $p = 0.03$, *). The interaction effect between avatar shape and avatar motion did not yield a significant difference ($F(3, 45) = 1.056$, $p = 0.4$, n.s.).

Regarding the ANOVA for humanness scores obtained from questionnaires in avatar shape conditions, the results indicated that the main effect of the avatar shape was highly significant ($F(3, 45) = 75.170$, $p < 0.001$, ***). Shaffer's multiple comparison test for humanness scores revealed the following results: HumanShape > AbbreviatedShape (*), HumanShape > ScatteredShape (*), ScatteredShape < AngularShape (*), AbbreviatedShape < AngularShape (*), HumanShape > AngularShape (*), and AbbreviatedShape = ScatteredShape (n.s.).

EOF

This is a provisional file, not the final typeset article
